# Power and positionality in the practice of health system responsiveness at sub-national level: insights from the Kenyan coast

**DOI:** 10.1186/s12939-024-02258-5

**Published:** 2024-09-02

**Authors:** Nancy Kagwanja, Sassy Molyneux, Eleanor Whyle, Benjamin Tsofa, Hassan Leli, Lucy Gilson

**Affiliations:** 1 Health Systems and Research Ethics Department, KEMRI-Wellcome Trust Research Programme, Kilifi, Kenya; 2 Centre for Tropical Medicine and Global Health, Nuffield Department of Medicine, Oxford University, Oxford, UK; 3 Health Policy and Systems Division, School of Public Health, University of Cape Town, Cape Town, Western Cape South Africa; 4 County Department of Health, Kilifi County Government, Kilifi, Kenya; 5 Department of Global Health and Development, London School of Hygiene and Tropical Medicine, London, UK

**Keywords:** Public feedback, Power, Responsiveness, Voice, Health system

## Abstract

**Background:**

Health system responsiveness to public priorities and needs is a broad, multi-faceted and complex health system goal thought to be important in promoting inclusivity and reducing system inequity in participation. Power dynamics underlie the complexity of responsiveness but are rarely considered. This paper presents an analysis of various manifestations of power within the responsiveness practices of Health Facility Committees (HFCs) and Sub-county Health Management Teams (SCHMTs) operating at the subnational level in Kenya. Kenyan policy documents identify responsiveness as an important policy goal.

**Methods:**

Our analysis draws on qualitative data (35 interviews with health managers and local politicians, four focus group discussions with HFC members, observations of SCHMT meetings, and document review) from a study conducted at the Kenyan Coast. We applied a combination of two power frameworks to interpret our findings: Gaventa’s power cube and Long’s actor interface analysis.

**Results:**

We observed a weakly responsive health system in which system-wide and equity in responsiveness were frequently undermined by varied forms and practices of power. The public were commonly dominated in their interactions with other health system actors: invisible and hidden power interacted to limit their sharing of feedback; while the visible power of organisational hierarchy constrained HFCs’ and SCHMTs’ capacity both to support public feedback mechanisms and to respond to concerns raised. These power practices were underpinned by positional power relationships, personal characteristics, and world views. Nonetheless, HFCs, SCHMTs and the public creatively exercised some power to influence responsiveness, for example through collaborations with political actors. However, most resulting responses were unsustainable, and sometimes undermined equity as politicians sought unfair advantage for their constituents.

**Conclusion:**

Our findings illuminate the structures and mechanisms that contribute to weak health system responsiveness even in contexts where it is prioritised in policy documents. Supporting inclusion and participation of the public in feedback mechanisms can strengthen receipt of public feedback; however, measures to enhance public agency to participate are also needed. In addition, an organisational environment and culture that empowers health managers to respond to public inputs is required.

**Supplementary Information:**

The online version contains supplementary material available at 10.1186/s12939-024-02258-5.

## Introduction

Health system responsiveness was introduced as a health system goal in the World Health Report 2000 and defined as ‘*when institutions and institutional relationships are designed in such a way that they are cognisant and respond appropriately to the universally legitimate expectations of individuals’ (pg 3)* [1]. Responsiveness is closely linked to the idea of public or community participation, introduced in the Alma Ata Declaration, and gaining renewed attention in efforts to build people-centred health systems [2, 3]. Responsiveness is, then a broad, multi-faceted and complex health system goal [4, 5]. Yet much of the responsiveness literature has focused on evaluating service delivery interactions with patients, reflecting a narrow perspective of responsiveness [5].

Given calls for a broader conceptualisation of responsiveness that adopts a system lens [4, 5], and as reported elsewhere (Kagwanja et al., submitted), we examined the organisational influences over the responsiveness practices of selected case study Health Facility Committees (HFCs) and Sub-county Health Management Teams (SCHMTs) operating at the sub-national level in Kenya. In many low- and middle-income countries (LMICs), these structures play critical governance roles (including managing public feedback) within the health system. Kenyan health sector policy documents also identify responsiveness as an overall health system goal [6–8], although few Kenyan studies have examined responsiveness [9, 10]. We conceptualised responsiveness as how the health system reacts/responds to public feedback [11]. The term *feedback* refers to the input, views and concerns raised by the public, while a *feedback channel* is the mechanism through which these views, concerns and inputs reach the health system. In our work we applied a framework that considers organisational capacity as comprising interacting features of hardware (funding, staffing, technology), tangible software (procedures, managerial skills) and intangible software (relationships, communication, power dynamics) [12].

Our initial analysis highlighted that little public feedback was picked up through the HFCs and SCHMTs; and for the feedback that was received few responses were generated (Kagwanja et al., submitted). SCHMT and HFC responsiveness practices were constrained by interacting system features: inadequate funding and staffing of feedback mechanisms; unclear procedures and guidelines for handling public feedback; norms and power dynamics (Kagwanja et al., submitted). In this paper we examine in more detail how power dynamics, specifically, interplay with and impact on all dimensions of system responsiveness. Our overall questions were: how does the exercise of power impact on system responsiveness, and what influences how power is exercised by the actors involved?

Power, defined as the ability to influence others’ behaviour or shape the course of events [13], is dynamic and can be discerned in interactions within organisations and in relationships between actors [14–16]. In this article, we sought to examine various practices of power, recognising not just top-down flows within a bureaucracy but also the reality that power is exercised by actors across health system levels, including those at the frontline of service delivery. Although power and positionality are thought to influence responsiveness [4, 5, 17], very little responsiveness research has examined power dynamics in any detail [18]. Adopting a power lens can extend our understanding of responsiveness by illuminating how and why actors receive and respond to public feedback, and findings from such research can be applied to support the design of interventions seeking to strengthen responsiveness. Such analysis also extends the still limited body of research considering power dynamics in health systems more generally [13].

### Context: changes in broader and health system governance

Kenya has had different forms of decentralised government systems since independence ranging from a federal system to local authorities and deconcentrated districts units [19–21]. However these decentralisation efforts did not adequately address the needs for inclusivity in public resource sharing, and peoples’ participation in public governance which coupled with the promise of a new constitution in 2002 and a contested election in 2007, created an impetus for devolution [22].

At independence, in 1963, the public health system was highly centralised around the national Ministry of Health which had responsibility for policy direction, coordination of government and NGO activities, implementation of service delivery, and monitoring and evaluation of policy changes [23, 24]. In 2013, devolution from a highly centralized national system in Kenya that had eight provinces and 80 districts to a decentralized governance system with 47 semi-autonomous counties [25] occasioned changes within the health system. Following devolution, the Ministry of Health has responsibility for health policy formulation, training and regulation of health services while county governments have responsibility for policy implementation and service delivery [25]. County Health Management Teams and Sub-county Health Management Teams (SCHMTs) provide oversight, manage and plan health service delivery at county and sub-county levels respectively [26]. This study was conducted in one of five coastal counties in Kenya.

Several of the objectives of devolution have implications for the health sector: promotion of democratic and accountable use of state power; acknowledging and recognising the public’s right to manage their own affairs and further their development; protecting and promoting the interests and rights of marginalised groups; and promoting the provision of easily accessible public services closer to the people [25, 27]. Prior to devolution, at the different levels of the health system, various mechanisms were introduced over time to promote public participation in health system activities. These include HFCs at dispensary and health centre levels, and hospital management boards. These mechanisms continued to be operational in the newly devolved context [28]. Literature suggests that reforms such as decentralisation and community participation bring formal oversight closer to the public and may encourage responsiveness to public needs and desires [17, 29]. Decentralised governance arrangements form the broader context of this study. A form of oversight that could enhance responsiveness to public feedback includes political positions introduced by devolution such as the Governor, Members of County Assembly, and politically appointed county government officials. We include interactions with these actors, particularly Members of County Assembly in our exploration of responsiveness practice across the HFCs and SCHMTs.

## Methods

### Study design

We adopted a case study approach because of its appropriateness for examining complex phenomena [30, 31]. The study design and data collection details are summarised in Tables 1 and 2 below.

**Table 1:** Study design and data collection details

Case definition and selection of cases
• In our study, ***a case is a ‘processing space’ within the health system where public feedback can be received, processed and responded to*** • SCHMTs and HFCs were selected as cases because of their varying characteristics and potential to be rich in information
• HFCs are comprised of community members, health facility managers, political and administrative representatives, while SCHMTs are mainly composed of health managers • SCHMTs are higher up in the health system hierarchy reporting to the County Health Management Team (CHMT) and oversee primary healthcare facilities including their linked HFCs • HFCs and SCHMTs provided an opportunity to learn about interactions across health system levels that impacted responsiveness to public feedback • We selected two SCHMTs within one county, and two HFCs per SCHMT. These numbers were informed by a need to allow for in-depth exploration within the available time and resources
**Data collection**
• Between June and December 2020, we conducted 35 in-depth interviews with Primary Health Care facility-in-charges, sub-county and county health managers, and Members of County Assembly, and four focus group discussions with HFC members (Table 3) • Members of County Assembly are local political representatives who have legislation and oversight responsibilities within the County [27] and serve as ex-officio HFC members • We also conducted observations of SCHMT activities and a review of SCHMT and HFC minutes (Table 3). Data collection was guided by interview and topic guides (Supplementary Material 1) and an observation checklist (Supplementary Material 2)

**Table 2:** Summary of data collected

Data collection activity	Details
In-depth interviews	Sub-County Health Management Team members (16)- County Health Management Team members (3)
	Health facility managers (5) and frontline workers (8)
	Members of County Assembly (4)-3 linked to HFCs A, B, & C; one member of the County Assembly Health Services Committee
Focus Group Discussions	HFC members (except in-charge) (4)
Observations of meetings	Observation of Sub-county Health Management Team meetings & support supervision (SCHMT-1) between July and August 2021 (6 meetings)
Document review	-County level documents-County Budget Outlook Paper; County Integrated Development Plan, Health Sector Mid-term Review, County Budgets)
	-Sub-County Health Management Team & Health Facility Committee minutes -Sub-County Health Management Team & Health Facility Annual Work Plans

**Fig. 1:**
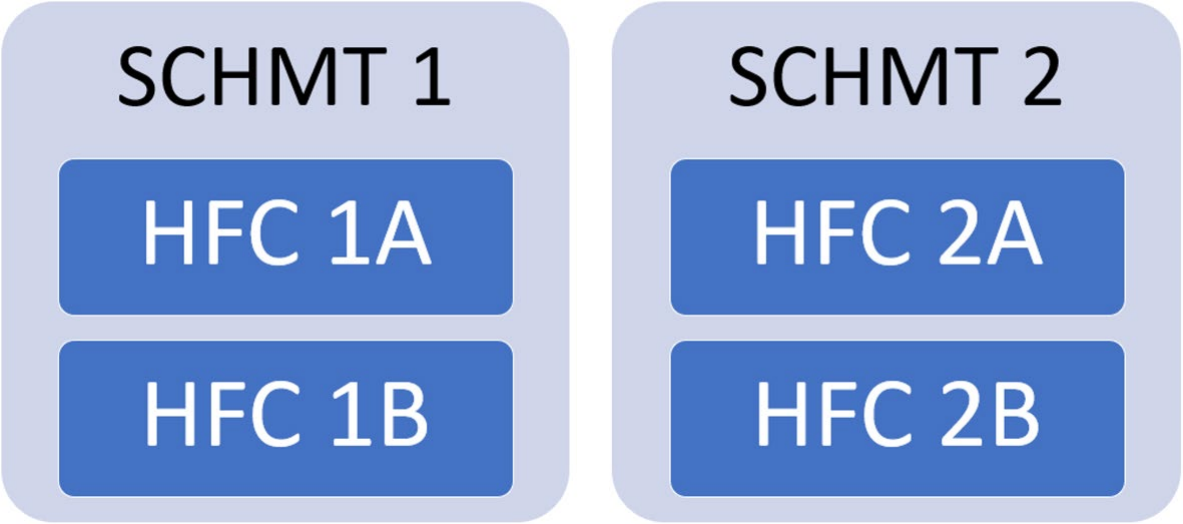
Cases for in-depth exploration

To protect confidentiality, the SCHMTs and HFCs selected are identified with numbers and letters (Fig. 1). The Primary Health Care (PHC) facilities linked to the case study HFCs are identified as Facility 1A, 1B, 2A and 2B.

The broader study that generated the findings analysed in this paper was conducted in two phases. The first phase began during the early days of the COVID-19 pandemic in June 2020 (about three months after the first case of COVID-19 in the county under study was reported). The second phase was conducted a year later (June 2021) as the pandemic continued to unfold, by which time much of the health systems functions had been restored to near normal. This approach helped to minimize the dramatic effects of the COVID-19 pandemic. The study findings reported here focus on the second phase, while the data collected during the early days of the COVID-19 pandemic were developed into a policy brief to inform the COVID-19 response at the time.

### Conceptual framework

In our analysis we applied concepts drawn from Gaventa’s power cube (Table 3) [32, 33] and Long’s ideas about actor interfaces (Tables 4 and 5) [14, 34]. Gaventa’s power cube was applied because it recognises levels of the system as influencing each other, such as the national, sub-national and facility levels in the Kenyan health system, as well as allowing for various exercise of power within and around the formal, closed, and invited spaces of HFCs and SCHMTs, and for the possibility of more informal spaces being claimed.

**Table 3:** Gaventa’s dimensions of power [32, 33]

Spaces for power	Details
Closed spaces	Decisions are made by actors behind closed doors. Within the state this might be in the form of elites, bureaucrats or elected representatives making decisions without involvement of the broader public
Invited spaces	Spaces are created into which the public (as users, citizens or beneficiaries) are invited to participate by various kinds of authorities such as governments, non-governmental organisations
Claimed spaces	Spaces formed by less powerful actors from or against the power holders. These may form following popular mobilisation, or around identity or issue-based concerns, or like-minded people coming together to debate issues
**Forms and visibility of power**	
Visible	Definable and observable decision-making. Includes formal structures of authority, institutions, and procedures of decision-making
Hidden	Certain powerful people and institutions maintain their influence by controlling who gets to the decision-making table and what gets on the agenda. Mainly operates by excluding certain people and devaluing the concerns of less powerful groups
Invisible	Shapes the psychological and ideological boundaries of participation. Significant problems and issues are not only kept from the decision-making table, but also from the minds and consciousness of the different players involved, even those directly affected by the problem. May be perpetuated by socialisation and cultural processes that define what is acceptable
**Levels of power**	
Global	Decision-making based on agreements and treaties by global and international bodies such as WHO, World Bank
National	Decision-making at the macro level, to include national governments and development partners
Local	Decision-making at the sub-national level, might include counties, districts, provinces down to the community level

**Table 4:** Actor lifeworlds [14, 34, 35]

	Broad Dimensions of Actor Lifeworlds		
	Relationships of Power	Personal Life Concerns or Characteristics	Social/Cultural/Ideological worldviews
Elements	Social positions or status, authority, organisational/ institutional hierarchy, technical/ professional expertise, resourcefulness, gender, caste, class relations	Individual interests, motivation, identity, image, recognition, previous experiences, cognitive and behavioral traits, situations in personal lives, understanding	Values, norms, beliefs, moral standing, religious views, organisational/ institutional norms and culture

**Table 5:** Power practices [35, 34]

Power practice	Definition
Domination	Certain actors holding positional power (managerial, professional) over other actors
Negotiation	Occurs when actors are partially aligned to another actor’s decisions or actions
Collaboration	Actors work together to support an action or decision
Contestation	Opposition between two actors interacting at an interface
Resistance	Actors object to or oppose a decision or action of another actor

Long’s actor-oriented perspective on power, meanwhile illustrates how the lived experiences of actors, their interactions and power struggles shape policy implementation. Actor interfaces are points of interaction between actors in relation to a policy. Power dynamics manifested at these interfaces are shaped by actors’ lived experiences, *actor lifeworlds* [14, 34], which are dynamic and dependent on an actor’s context. They include knowledge and power relationships in society and organisations, personal characteristics, and worldviews (Table 4). Power practices ranging from domination, collaboration, negotiation, resistance to contestation (Table 5) may be observed within actor interfaces [14, 35].

### Data analysis

Transcribed data were imported into NVIVO12 and analysed using a framework approach, given the policy and practice orientation of this work [36]. We coded for data on actors with whom SCHMT and HFC members interacted when receiving and responding to feedback; the spaces and levels where feedback was received, discussed, and responded to; forms of power observed across and within SCHMTs and HFCs; power practices by individuals or groups of actors and their effects; and actor life-worlds underpinning practices of power. Data for actor life-worlds were obtained by coding for actor life-world dimensions and then sub-coding for the characteristic elements of the life-worlds described in Table 4, an approach that has been used in other studies that have drawn on actor interface analysis to examine power [18, 37, 38]. We examined the coded and charted data to develop categories and themes, and to find associations that could support abstractive interpretation. This process was guided by the research questions and involved finding linkages between the emerging findings and existing literature.

## Results

Our findings suggest that far from being a linear process of sharing feedback up-wards from the public to the HFCs and then to the SCHMTs responsiveness practices were complex; there were interactions across multiple actor interfaces (Supplementary Material 3) some of which included informal interactions. For example, the public bypassed either or both the HFCs and SCHMTs to form interfaces with higher-level health system actors or others in the broader public sector who in turn sometimes shared the feedback down the health system for response at SCHMT or HFC level or responded themselves with varying effects on system-wide responsiveness.

We present the actor interactions and power dynamics underpinning these observed responsiveness practices in two broad sections, the first considers how the exercise of power impacted responsiveness. The second explores what influenced how power was exercised by actors in relation to responsiveness. For each of these sections we consider separately how power influenced i) receiving and ii) responding to public feedback.

### How did the exercise of power impact responsiveness?

#### Exercise of power and its effects on receiving public feedback

For the most part, the exercise of power constrained the public from sharing feedback, undermining responsiveness. As Table 6 shows, in three interfaces (Rows 1–3), a combination of hidden and invisible power enabled the practice of power as domination, with negative impacts on receiving feedback. In these interfaces, such power shaped the composition of HFCs by excluding vulnerable groups, such as women and youth, and limited the functionality of feedback mechanisms by denying them adequate resources. At the same time, in three interfaces (Rows 5–6), visible power, reflected in the practice of power as domination and control, reinforced the constraints to receiving public feedback. Specific examples are presented below to illustrate these experiences.

**Table 6:** Forms and practices of power and their effects on receipt of public feedback

	**Actor interface**	**Exercise of power**		**Observed effect on responsiveness element**
		**Forms of power**	**Practice of power**	
1	Public/health managers/health providers	**Invisible power** expressed as: - low confidence and lack of knowledge on how to share feedback by members of the public - socio-economic concerns among members of the public and among vulnerable groups - organisational norms among health managers (SCHMT members) who were focused on service delivery indicators, and paid little attention to public feedback channels	**Domination** of the public	- Hindered receiving of public feedback by HS actors -Undermined functioning of public participation mechanisms by keeping members of the public from attending public participation - Poorly functioning, feedback mechanisms that might have picked up public feedback were not set up, limiting the amount of public feedback that was received
2	Public/HFC	- **Invisible power** expressed in an organisational culture of electing only those that attended public meetings - **Hidden power** expressed in restriction of elections of HFC members to village elders and/or household representatives	**Domination** of vulnerable groups	- Vulnerable groups commonly absent from these meetings were excluded from participatory feedback mechanisms - HFC had no youth, PLWD representation and few women representatives - The absence of vulnerable groups from participatory feedback mechanisms limited the range of feedback available through HFCs
3	Department of Finance/ SCHMT/ public	The Department of Finance exercised **hidden power** over the SCHMT and public by limiting the duration of public participation meetings to one day	Department of Finance **dominated** the SCHMT and public during public participation meetings	Limited public input on planning, and budgeting for the health sectors
4	Department of Finance/SCHMT/HFC	Department of Finance exercised **visible power** by using organisational position over SCHMT to re-allocate funding for HFC training to supplier payments	**Control and domination** of SCHMT activities by Department of Finance	Challenges with HFC functioning due to inadequate training
5	Public/ Department of Finance /SCHMT	The public pushed back against the Department of Finance use of their **visible power** to limit time available for the public to share their views on budgets	Public **resistance and contestation** with finance and health department managers over little time for engagement	Discontinued the public participation meeting, hindering any inclusion of public views or input into the budget
6	Public/MCA/HFC and Public/MCA/SCHMTs	The public leveraged the **visible power** of political actors and senior county officials by bypassing SCHMTs and HFCs to share feedback	**Contestation** between public and SCHMTs and HFCs over informal feedback to politicians and county officers	Facilitated entry of public voice into the public health sector

*Abbreviations*: *HFC* Health Facility Committee, *HS* Health System, *PLWD* People Living With Disability, *SCHMT* Sub-county Health Management Team

Invisible power, manifested in the public’s limited understanding of health system functioning, concerns about victimisation and a lack of confidence in sharing feedback, enabled their domination by other health system actors. As noted by one county health manager:

*“The level of confidence is very low, yes because it is through public forums that…the public like talking, where they are many so they know if they talk, this other people will assist me but [when a member of the public is] alone... it’s very hard, you [the health manager] hear rumours, but when you try following up, they don’t open up” (County Health Manager-002)*

Similarly, invisible power, as manifested in organisational norms of electing only those who showed up at the chief’s *baraza* (community-wide meeting within a local area), enabled domination by some actors during elections and resulted in the exclusion of vulnerable groups (such as youth and People Living With Disability) from HFC membership. Several HFC members held the view that ‘*the youth ignored attendance of these meetings, and so were responsible for their own lack of awareness about health service issues*.’ However, socio-economic concerns deterred the youth from attending *barazas*, which were held during the day, when most youth were working at jobs that paid a daily wage.

Hidden power also interacted with invisible power in the HFC member selection process to support the domination of vulnerable groups and undermine equity in responsiveness. In HFC-2A, respondents reported selecting HFC community members from among pre-existing village elders and *nyumba kumi* (cluster of ten households) representatives. This approach excluded youth as the roles of village elder and household representatives were mainly occupied by middle-aged men. The HFCs did not meet the mandated quotas for vulnerable groups, for example only two HFCs had youth representatives, while there were no representatives of People Living With Disabilities across all the HFCs under study (Kagwanja et al., submitted).

The exercise and effects of visible power were, meanwhile, reflected in SCHMT and public interactions with the County Department of Finance. By limiting public participation meetings to a single day for multiple departments within the county government, the finance department practiced power as domination. Public concerns and questions were often cut short, and requests by SCHMT members to extend the duration for public participation were reportedly met with the response that *‘resources for public participation were only allocated for one day’.* At the SCHMT/County Department of Finance interface funds allocated for HFC training were also often used for purposes deemed more pressing, as expressed by one SCHMT respondent:

*“I put up the request [for HFC training] it goes through the processes to the treasury then when it reaches the treasury there is no money. You wait for like over a year in fact that money [for training] may not come at all so that is the biggest challenge because any time money arrives at the treasury they have other priorities like people the suppliers have not been paid” [SCHMT1-009]*

As a result, many of the community members had not received training on what was expected of them in their roles as representatives in the HFC. SCHMT members who were responsible for training them acknowledged this gap, noting that members joining the HFC from the last two election cycles were not comprehensively trained on their roles because SCHMTs experienced challenges in accessing funding for this activity (Kagwanja et al., submitted).

In addition, as shown in interface 5 of Table 6, even when the public pushed back against the finance department, resisting the exercise of their visible power, the ultimate impact was to limit feedback, as public participation meetings were discontinued.

Finally, Table 6 shows that in one interface (row 5) the public’s practice of power as contestation and resistance enabled the giving of feedback. In this case, the public leveraged the visible power of other actors by using informal mechanisms to share feedback. We judged this as claiming space (Table 7). These informal mechanisms, ranging from social media, informal calls to local politicians and high-ranking county officials, and use of mainstream media, all provided an alternative avenue to the formal spaces of SCHMTs and HFC, respectively, for the public to voice concerns.

**Table 7:** Characterisation of case study spaces for processing feedback drawing on Gaventa’s spaces and levels of power

Space	Characterisation drawing on Gaventa’s spaces of power	Levels where power was exercised
SCHMT	Closed space-comprised health managers only who received, discussed, and took action about public feedback without involving members of the public in decision making	Across sub-county and county levels
HFC	Invited space-The public were ‘invited’ by H/S actors (government) to participate in decision-making on PHC facility issues –financial management, and link between the public and PHC facility	At the local facility level
Informal feedback channels-direct calls to senior health managers, county officials, social media	Claimed space-These were utilised by the public who bypassed the SCHMTs, HFCs and PHC facility staff to ‘leverage a response’ from the health system	Cut across multiple levels as members of the public engaged senior health managers, county officials and political actors who had linkages to the local, sub-county and county levels

*Abbreviations*: *HFC* Health Facility Committee, *PHC* Primary Healthcare, *HFC* Health Facility Committee

### The exercise of power and its effects on responding to public feedback

Table 8 highlights that for responding to public feedback, the exercise of power both supported and constrained responsiveness. The positive effects of exercising power, shown in interfaces 1 and 3–6, included strengthening relationships that contributed to functioning of feedback mechanisms, and enhancing access to resources which were useful to support system responsiveness. In contrast, the negative effects, as shown in interfaces 2, 7–10, included strained relationships between actors, limiting information necessary for the generation of responses and orienting health providers and managers away from public feedback. Specific examples are again presented below to illustrate these experiences.

**Table 8:** Forms and practices of power and their effects on responding to public feedback

	**Actor interface**	**Exercise of power**		**Observed effects**
		Forms of power	Practice of power	
1	HFC/Facility manager	HFC members exercised **visible power** by leveraging their facility oversight mandate to negotiate for rescheduling of staff meetings to afternoons to reduce waiting time, to discuss HCW behaviour towards patients in all four facilities, and to approve purchase of drugs using facility-level funds in facility 2A	HFC members **negotiation** with and **facilitation** of facility-in-charge for local generation of responses	HFC member’s exercise of power tilted power dynamics in favour of service users resulting in services more aligned to patient needs
2		HFC members (Facility 2B) exercised **visible power** from formal mandate for oversight to report negative HCW conduct to in-charge; Facility managers exercised **visible power of** managerial position in addressing issues raised by HFC with staff	**Tensions and contestation** in Facility 2B over perceived slow and ineffective responses by facility-in-charge to feedback about poor HCW conduct	Strained relationship between HFC and Facility staff (including facility-in-charge) undermined HFC functioning of the HFC
3	HFC/MCA	MCAs exercised **visible power** to generate responses to public feedback received from HFCs by availing requested drugs and supplies and lobbying the County Executive for inclusion of resource-intensive public priorities in County Budget	MCAs **facilitation** of access to needed supplies for service delivery	Enhanced access to resources and generation of responses at facility-level resulting in services aligned to patient needs
4	HFC/NGOs; HFC/ private businesses	HFCs exercised **visible power** by mobilising resources from NGOs and private businesses through formal requests for purchase of drugs, and to fill staffing gaps	HFC **facilitation** of access to resources for service delivery	
5	SCHMT/HFC/Facility staff	SCHMTs exercised **visible power** to respond to public feedback related to poor HCW conduct	SCHMTs **dialogue and mediation** with HCWs about whom negative public feedback was shared	
6	SCHMT/MCA	SCHMT-1 and MCAs exercised **visible power** to lobby MCAs to engage with CHMT to generate a response	SCHMTs **negotiated** with (MCAs) political actors for resources	
7		SCHMT-2 exercised **visible power** to deny MCAs requests for members of the public connected to them to be prioritised for service	**Control of SCHMTs** on service delivery procedures	Strained relationships between SCHMT members and MCAs
8	SCHMT/CHMT	CHMT exercised **visible power** over SHCMT by failure to communicate rationale for actions and limiting SCHMT access to information	CHMT **domination** over SCHMT	Limited SCHMTs access to information undermining functioning as a feedback mechanism
9	SCHMT/CHMT	CHMT exercised **hidden power** over SCHMTs by not providing timely access to budgets to SCHMT members	CHMT **control** of information	
10	Public/SCHMT/CHMT	**Hidden power**- higher level HS actors ‘removed’ issues of medical negligence from discussion by SCHMT and with the public	**Domination** of the public by HS actors in matters of perceived medical negligence	Oriented health managers away from public feedback
11	Public/health providers/health managers	**Invisible power**-Organisational culture of defensiveness when the public raised concerns about medical negligence	**Domination** of the public by health managers and health providers	

*Abbreviations*: *CHMT* County Health Management Team, *HCW* Health Care Worker, *HFC* Health Facility Committee, *HS* Health System, *MCA* Member of County Assembly, *PHC* Primary Health Care

The positive effects on responsiveness were linked mainly to visible power and power practices such as negotiation, collaboration, and facilitation, at the HFC/Facility manager interface (row 1), and dialogue and mediation at the SCHMT/HFC/facility staff interfaces (row 5). All appeared to foster positive working relationships and resulted in the generation of responses and service delivery more aligned to public needs. However, at these same interfaces we observed that visible power also had negative effects on responsiveness. For instance, at the HFC/Facility manager (row 2) and the SCHMT/Member of County Assembly (row 7) interfaces we observed strained relationships arising from contestation and control respectively, and these negatively impacted HFC and SCHMT functionality in generating responses to public feedback.

Though at play less often, hidden, and invisible power also constrained response generation by enabling control and domination of the SCHMT and the public by other actors (interfaces 9–11). For example, hidden power exercised by the County Health Management Team in controlling budget information limited SCHMTs’ capacity to respond to public concerns about priority-setting:

“*My attendance [as a SCHMT member] is not consistent, it is not a guarantee that you will always be invited [to the public participation meeting] ... these budgets once they are already done, they are done, going back to the drawing board it’s expensive. Now redoing it, it’s not very easy like I have told you most of the time it’s like a ceremony, you see this is what we have done, so maybe their [the public’s] suggestions do not count much yes, they [the public] have to swallow it the way it is” (SCHMT2-006, author emphasis)*.

The public’s domination by health managers, meanwhile, was enabled by the exercise of hidden power when issues related to patient deaths due to perceived negligence were simply not discussed. As one SCHMT member described:

*“You know in our setting we lose so many patients in the line of duty and it’s unfortunate. Had it [patient death due to perceived medical negligence] happened in a private facility, maybe it would have warranted an explanation, but in our public facilities, that never happens. As staff in the facility, we are not allowed to communicate externally. So, we escalate the issue to the department, and then if it’s a public apology or explanation then it comes from the department not from the facility.” (SCHMT1-05)*

Within interface 1, we also identified invisible power as reflected in perceptions among health providers and managers that the public did not understand health system functioning and medical procedures, while health providers were ‘*experts who commonly dealt with life and death issues’ (CHMT-01).* We judged these views to reflect invisible power manifesting in an organisational culture of defensiveness as further illustrated below:

*“Like for example when interrogating people that were on duty that day [when a mother had a stillbirth delivery perceived by the public to be due to medical negligence], most of them would ask you, we had 21 deliveries that day, what makes this one unique? Was it because her baby passed away and everyday babies are passing away in the maternity so, it makes it . . . it looks like a normal occurrence that occurs…that is unpreventable.” (CHMT-02)*

### What influenced the exercise of power?

In this section we explore the drivers of exercises of power referencing the lifeworld analysis in Tables 9 and 10. The lifeworlds presented here are linked to power practices at interfaces highlighted in Tables 6 and 8. Thus, in this section we identify connections between actor lifeworlds and exercise of power and highlight patterns across processes of receiving and responding to public feedback.

**Table 9:** Actor lifeworlds underpinning exercise of power in receipt of public feedback

	**Power practice at Actor interface and effect on responsiveness**	**Underpinning actor lifeworlds**		
		**Positional power relationships**	**Personal concerns or characteristics**	**Social, cultural ideological worldviews**
1	Domination of the public by health managers and health providers undermined receiving public feedback		-Public’s previous negative experiences with HCWs -Concerns among members of the public about victimisation after sharing feedback	- Health managers’ and providers’ belief that public feedback was subjective and incoherent - Health managers’ and providers’ belief that public had low understanding of health system functioning -Social status and respect accorded to healthcare workers by members of the public - Organisational norm among health managers and providers of prioritising service delivery indicators
2	Domination of the public and SCHMT by the Department of Finance undermined receiving public input on health sector budgeting and planning	Organisational power and budgetary control of Department of Finance		
3	Domination of vulnerable groups during HFC members selection excluded youth and people with disability from HFCs		-Many youth constrained from attending chiefs barazas by work that paid daily wages (personal situations)	-Organisational culture of electing only those that attended chief’s barazas
4	Members of the public bypassing HFCs and SCHMTs to share informal feedback with political actors provided an avenue for public feedback (informal feedback)	Public’s social connections to political actors and senior county officials	Political actors and political appointees’ interests to appeal to voter base	Public’s belief in their right to air grievances
5	Contestations between the public, HFC and SCHMT members when the public share feedback directly with political representatives	Political actors had oversight responsibilities over health system managers	Health facility committee members (particularly ex-officio members) and sub-county health managers concerns about their and facility/department reputation	Public’s belief in their right to air grievances
6	Public resistance and contestation with department of Finance at public participation meetings where their concerns were not addressed ultimately locked out public input	Organisational power and budgetary control of Department of Finance		Public’s belief in their right to air grievances

*Abbreviations*: *HFC* Health Facility Committee, *SCHMT* Sub-county Health Management Team

**Table 10:** Actor lifeworlds underpinning exercise of power in responding to public feedback

	**Power practices at actor interface (Positive and negative Responsiveness impact**	**Actor lifeworlds**		
		**Positional Power relationships**	**Personal concerns/characteristics**	**Socio-cultural ideological worldview**
1	HFC members negotiation with facility manager and facilitation of access to resources **supported generation of responses** i.e. improved waiting times & improved drug availability at facilities	HFC members formal mandate for facility oversight	HFC members commitment to ensuring continued service delivery	Organisational norms about public sector working hours
2		HFC members mandate for oversight of facility-level funds		HFC-2A members’ beliefs that specific categories of drugs (antihypertensives, diabetes drugs) should always be available
3	Tension and contestation between HFC members and facility in-charge, **strained relationship and undermined HFC functioning**	Facility-in-charge position of authority; HFC members’ formal mandate for facility oversight	-HFC members’ frustration over perceived ineffective responses to complaints about HCW conduct -Facility manager and staff discomfort with confrontational HFC monitoring	Facility manager and staff’s belief that HFC had a responsibility to defend facility against bad publicity -HFC members’ suspicion and mistrust of facility managers and staff in HFC—2B
4	Collaboration, facilitation between MCAs and HFC members **facilitated generation of responses**	-HFC mandate for oversight over facility service delivery; MCA political power and access to resources	-HFC members’ commitment to continued service delivery -MCA personal interests to appeal to voter base;	
5	Collaboration, facilitation between HFCs, NGOs and private businesses **supported generation of responses**	-HFC mandate for oversight over facility service delivery -NGOs access to resources		
6	SCHMT mediation between facility staff and HFC supported** generation of responses**	Managerial authority of SCHMTs over PHC facility staff	-Commitment to continue service delivery -Concerns about safety of HCWs	
7	Collaboration between SCHMT, MCA to address public feedback,** supported SCHMT to generate responses**	-SCHMTs’ managerial power -MCAs’ positional power and oversight responsibility for budget approval	Personal interests of MCAs to get appeal to their voter base	
8	Contestation between SCHMT and MCA over requests for prioritisation of well-connected public **strained SCHMT-MCA relationship**	-Managerial authority of SCHMTs over facility staff; -MCAs’ positional power and oversight responsibility for budget approval		SCHMTs views about justice and fairness in service delivery
9	Control of information at SCHMT/CHMT interface u**ndermined SCHMT capacity to respond**	Managerial authority of CHMT over SCHMT SCHMTs low access to information about consolidated health sector budget	-SCHMT experiences of receiving little information and clarification for CHMT actions	-Organisational norm of sharing information up-wards and CHMT perception that SCHMT were part of the health system and therefore were aware of system problems
10	Domination of the public by health system actors in matters of perceived medical negligence **undermined responsiveness**	Higher level county officials positional authority over CHMT and SCHMT; Professional position of health managers and health providers; Low knowledge of members of the public	Health managers, and health providers self-perception as experts while the public were viewed as non-experts who did not understand medical procedures, and health system functions	Organisational norm of not admitting liability

*Abbreviations*: *CHMT* County Health Management Team, *HCW* Health Care Worker, *HFC* Health Facility Committee, *HS* Health System, *MCA* Member of County Assembly, *PHC* Primary Health Care

Tables 9 and 10 show some differences in the elements of lifeworlds that influence the exercise of power between receiving and responding. For example, Table 9, illustrates that *social, cultural ideological worldview*, manifesting in beliefs and mindsets of the public and health managers and in organisational norms, were the main drivers of power practices that influenced receipt of public feedback. Both Tables 9 and 10 re-emphasise some of the points already raised in the previous sections concerning organisational power and norms reflected in visible and invisible power respectively. Notably, Table 9 shows the public believed they have a right to air grievances – and exercise their agency – but were thwarted by organisational and political power; whilst Table 10 re-emphasises the complex forces shaping responding (multiple actors, interactions, exercises of power and lifeworld elements). From Table 10, *positional power relationships* within organisations were the predominant driver of the exercise of power in responding to public feedback, backed up by *personal concerns* and *worldviews* across both SCHMTs and HFCs.

The public’s belief in their right to share feedback, though observed multiple times, was not overall, however, sufficient to support responsiveness. In one instance (Table 9, row 4) where it appeared to enable receipt of public feedback it was reinforced by other actor lifeworlds linked to politicians. These lifeworlds included: a desire to appeal to their voter base, reflecting politicians’ *personal concerns* with maximising their chances of being (re)elected, and the organisational *power relations* manifested in the authority of political representatives and senior county officials to whom health system actors were indirectly accountable.

However, as shown in Table 9, row 5, despite acknowledging the public’s right to share feedback, there were tensions around the public’s use of informal feedback mechanisms. Contestations against informal feedback were underpinned by *personal concerns* about the image of the department (SCHMT-1) and a desire to observe protocol (HFC-1A), and maintain a positive image with superiors:

*“But we don’t want them [the public] to go to the media, we don’t want them to go to Facebook, to Whatsapp and Twitter. It is a way of communication, yes, but let them come to us, we shall listen, because when they [the public] go to the media, Facebook, Whatsapp...okay it creates a lot of concern, a negative picture to the department and we do not want to look like we are not working (SCHMT1-001)*

Table 9 also shows that the public’s agency varied depending on the space where they interacted with other actors. For example, as already discussed, the youth’s need to work limited their engagement in responsiveness mechanisms (row 3). Moreover, as shown in row 1, within the health system, the public’s *personal concerns* about victimisation contributed to self-censorship (invisible power) as described below:

*“It is like there is a code that people have, see no evil hear no evil, very few report, often the complaints that you hear come from someone who is probably new in the community, or an outspoken person. Only 1 or 2 or 3 people will raise an issue but when you ask now, is this true…in a public forum, you will get surprised on how many people have gone through the same thing in the past and they have never reported” (CHMT002)*

*“There’s that fear of reporting, first because you don’t know who to tell, so we can say lack of knowledge about who to tell, then even if you know, you don’t know how they will take it, then thirdly there’s fear because if you say a healthcare worker did something to you, you don’t know if when you go to the facility you will be served or they will fail to serve you.” (HF2A-003)*

Table 10 highlights that sometimes one set of actor lifeworlds interacted to underpin power practices that in one situation supported, and in another, constrained responsiveness to public feedback. This is consistent with earlier findings about visible power being associated with both positive and negative power practices. For example, organisational *power relationships* reflected in the political power of MCAs (visible power), and MCAs’ *personal concerns* about winning elections interacted to support responding to public feedback in certain instances (row 7). A SCHMT-1 member reported:

*“For me I do a report, facility A needs a delivery room, at budgeting level, the executive then decides, the money is not enough so do we prioritize facility A or B, you know the county is vast, and because of resources, we also need a political push. That is why I call MCAs and say this will help you and will help the people. So, help me make this feasible…can you put money from your kitty or can you come and push the department. So, we must also, not play politics but engage because I want a delivery room which will make things much better. I am thinking of my people. I employ those tactics; I am not going against my bosses I’m just trying to get things done” (SCHMT1-007)*

However, these same lifeworlds (politicians’ positional power and personal concerns) also underpinned contestation and strained the SCHMT/MCA relationship (row 8), with the effect that MCAs were not valued for their representation role by SCHMT-2 who perceived that MCAs interfered with service delivery, ‘*did not follow protocol’*, and that their (the MCAs’) oversight should be carried out at the County Assembly and not in health facilities. SCHMT-2 members reported failing to act on public feedback shared via MCAs because they perceived that politically connected members of the public expected preferential treatment, revealing tensions around the exercise of political power. SCHMT-2 exercise of power in their interactions with MCAs was also influenced by managers’ *worldviews* reflected in the belief that public health service delivery should be fair to all:

*“Those people who are highly connected normally call influential people complaining of delays, but as service providers we are not supposed to discriminate based on position, financial or economic status. We should treat people equally, so you cannot let a person because he is connected to some big individual pass the queue while a mother who came as early as 6 a.m., has queued the whole day, it’s not justice”* (SCHMT2-003)

Although represented least frequently amongst the lifeworlds, Table 10 illustrates some examples of worldviews that shaped actors’ exercise of power to constrain generation of responses. This included organisational norms related to reluctance to admit liability and upward flow of information. Reluctance to admit liability can be linked to previously discussed organisational norms of viewing the public’s understanding of medical care and health system functioning as limited (invisible power). Concerning information flows, one SCHMT-A member noted:

*“...one thing that we have been lacking as a department I am sorry to say, we (the SCHMT) take our complaints [to the CHMT] but we don’t get feedback that this can be acted on, and this cannot, and why it cannot be acted, we need to get that feedback,” (SCHMTA-01)*

Among SCHMT members, there was a prevailing sense that sharing public feedback upwards *‘slowed or did not generate responses’* (Table 11) and was an invitation to have directions *‘dictated to them [the SCHMT].’*

**Table 11:** Organisational norms of up-ward information flow and limited down-ward flow of communication

The slow generation of responses and lack of clarity in rationale for action at CHMT level was illustrated by a recommendation by SCHMT-1 (in response to public complaints) to resume services that had been shut down in Facility-1A, which operated as a COVID-19 isolation centre. At the time of data collection, only HIV care and treatment services had resumed. The public served by Facility-1A had to seek care elsewhere. The CHMT and senior county-level decision-makers prioritised national-level guidance that required additional infrastructure to separate COVID-19 infected patients from the public seeking other outpatient services before re-opening all services. SCHMT-1 respondents reported lack of information on why the construction of this infrastructure had not been prioritised to support the resumption of all services. Facility-1A had stopped offering services to the public in November 2020, and only resumed in November 2021, following a decline in numbers of COVID-19 patients who required isolation	

In one facility, HFC members’ *worldviews*, reflected in an atmosphere of mistrust and suspicion of the facility manager and staff, underpinned contestation at the HFC/facility manager/staff interface. Tensions around this interface in Facility-2B, were underpinned by the staff’s *worldview* that the HFC ought to defend facility staff against negative public feedback rather than being *‘quick to fan the fire’*. Facility staff (and their SCHMT supervisors) perceived that health providers were unfairly held responsible for negative incidents and patient outcomes when these were related to drug stock-outs and staff shortages, factors, beyond their control. Table 12 below demonstrates how this contestation strained relationships between HFC members and staff (including the facility manager) and, overall, undermined responsiveness.

**Table 12:** Tensions and contestation in HFC-2B in reaction to frustrations of unresolved public feedback

In Facility-2B despite frequent dialogue with the facility-in-charge, HFC-2B members, felt that many of the responses (particularly those related to complaints about HCW conduct) were ineffective. At one point, interactions between the HFC chairperson and facility-in-charge deteriorated so much that the chairperson declined to sign HFC minutes. The HFC minutes supported a change of signatory from the outgoing facility manager (who had been promoted to the SCHMT) to the new facility manager in the facility’s bank account. The stalemate between the HFC community members and facility-in-charge over the change of signatory led to delays in the facility’s access to funds, including for paying support staff salaries despite there being money in the facility account. The impasse was later mediated by the SCHMT who engaged the HFC chairperson, staff, and facility-in-charge in dialogue. However, the overall effect was a strained relationship in which HFC-D was perceived by the facility staff as ill-prepared to carry out their functions, while the HFC perceived the Facility D staff (including their in-charge) as un-responsive, all of which damaged the HFC/health provider relationship and undermined the functioning of the HFC as feedback mechanism, and responsiveness to public feedback overall	

## Discussion

This study adds to the wider health system responsiveness literature that is limited by a predominant focus on service-level feedback, little attention to multi-level dynamics of receiving and responding to public feedback, and few theory-driven empirical studies [5]. Our findings reflect the complexity, multiple actors and interactions entailed in receiving and responding to feedback. Across the HFCs and SCHMTs flow of public feedback was not always direct and actor interfaces formed and re-formed resulting in power struggles that predominantly undermined responsiveness. In this section, we discuss the forms and practices of power, and their underlying actor lifeworlds including tensions arising from the exercise of power and consider how health system responsiveness could be strengthened based on our study findings.

In this study the public unwittingly excluded themselves from sharing feedback across multiple feedback channels, limiting how much input the health system received through HFCs and SCHMTs. Similar findings were reported in Ugandan and Kenyan studies which identified poverty as a structural issue that kept the public away from participating in priority setting [39, 40]. In our study, the youth and many workers, whose behaviour several HFC members and political representatives interpreted as disinterest, were pre-occupied with meeting socio-economic needs. Further, utilisation of feedback mechanisms by the public was also undermined by concerns about victimization and distrust of the system. These findings are consistent with studies from other LMIC contexts [41–44] and reflect the influence of invisible power in sustaining inequity in participation in feedback mechanisms.

Further, structural power in the form of organisational hierarchy constraining responsiveness was a dominant theme cutting across both receipt of and responding to public feedback. For example, due to their organisational position, HFCs predominantly generated responses to local-level issues for the short-term. SCHMTs also often had limited capacity to support feedback mechanisms (including HFCs) to function well and to generate responses. SCHMT members expressed feelings of disempowerment linked to their narrow authority to act, because of domination by the CHMT, senior public sector actors, political representatives, or national-level directives. Several studies have reported similar limitations that included domination by more powerful actors at HFC level [43, 45] and at district level [46–48]. Despite being less powerful relative to other actors, our case study HFCs and SCHMTs exercised some power to generate responses to public feedback ranging from one-off actions to measures s required follow-up and multiple actions. Even though these instances were few, they reflected actor agency and the skill to manoeuvre the organisational hierarchy.

The lifeworld analysis deconstructed actor agency, examining the motivations underpinning different power practices and highlighting tensions around exercises of power. One such tension centred around informal feedback and the exercise of political power—underpinned by the differing lifeworlds of the public, health managers and political representatives. Some literature suggests that informal feedback mechanisms are limited in building responsiveness [39, 40], but we observed mixed effects of informal interactions at the public/political actor interface. For example, issues that required one-off local responses could be resolved through informal mechanisms involving political representatives, however political power exercised by the Member of County Assembly appeared insufficient for persistent issues or those cutting across multiple facilities. This suggests that politicians stepping in to purchase supplies for facilities was not sustainable and is undesirable in the long-run as it interferes with strengthening systematic and system-wide procedures.

We also found a likelihood of selective responsiveness at the health managers/political representative interface, raising equity in responsiveness concerns. While politicians could generate responses for some forms of feedback, they also reportedly sought unfair advantage for those that were connected to them because of their interests to appeal to their constituents. Yet, these same political actors are needed to respond to the issues related to resource allocation often decided higher up in the health system and within the broader public sector, as shown in our study by interactions at the political actor/SCHMT interface. Support by political leadership has been demonstrated to be key in achieving broader health system reform and national-level health politics [49]. Our study findings suggest that relationships with political actors at the sub-national level are equally important but require careful management, given concerns about inequity in responsiveness when political actors favour certain segments of the population above others who might have greater needs.

The lifeworld analysis highlights tensions around HFCs’ functioning, specifically, around i) how HFCs balance the interests of the public and those of staff and ii) representation of vulnerable groups. Concerning the former, George et al. suggest that if HFCs serve as a way for the public and elected members to target healthcare workers as scapegoats for wider health system shortcomings then healthcare workers may withdraw their support of HFCs [50]. In our study the adversarial interactions between HFC members and frontline staff in HFC-2B, underpinned by differing worldviews on HFC roles, contributed to feelings of unfair treatment among staff, whilst some SCHMT members viewed HFC-2B members as *‘unprepared to do their roles’*, simply waiting for their tenure to expire. Yet support from facility staff and health managers is important for generating responses to feedback received through the HFC.

Concerning representation, our case study HFCs seemed to be an ineffective conduit for feedback from vulnerable groups. This was linked to the selection process that did not account for structural factors that kept vulnerable groups away during elections into HFC positions. Loewensen et al. highlight the tension around membership HFC membership [45]. On one hand, representatives of vulnerable groups bring the experience and voice of those with greater health needs to planning and organisation of service delivery. On the other hand, influential members of the public may be better able to address the power differences in the interaction between the public and healthcare workers [45]. Abimbola et al. argued that HFCs in Nigeria served many of their roles without being representative of marginalised groups, and that in contexts where HFCs receive little support from government or NGOs, elite members can use their resources and influence to achieve HFC goals [51]. However, Lodenstein et al. reporting on HFCs in West and Central Africa, acknowledged bias in representation with HFCs having more elite members, potentially limiting the range of feedback that could be received [52]. A similar point is made in a report that highlights how the absence of certain People Who Inject Drugs, who are considered a key population in HIV management in the Country Co-ordinating Mechanisms of the Global Fund, has often led to little inclusion of their input into country-level grants in effect undermining the delivery of comprehensive harm-reduction services [53]. However deliberate efforts to increase the diversity of key populations and include People who Use Drugs in the Country Co-ordinating Mechanism contributed to more targeted investments in key population programming [53, 54].

Based on our findings, to strengthen responsiveness, efforts need to be targeted at how invited spaces for receiving and responding to feedback are constituted and supported to continue functioning, as well as at the processes of receipt and response generation within both closed and invited spaces (considering actors’ agency and lifeworlds as well as structural forms of power). Concerning membership, the findings from our research suggest that both representation among, and the influence of, HFC members are important. Thus, membership in HFCs and in other invited spaces needs to be carefully balanced to ensure representation of vulnerable groups, while ensuring that there are members who are influential enough to reduce power asymmetries between the public and healthcare workers. To achieve adequate representation requires broad awareness building among the public, and consistent support from higher-level supervisors—for example by clarifying the rationale for mandated quotas for vulnerable groups and being present during community elections. Beyond ensuring their presence in these organizational structures, to build their agency, vulnerable groups could be motivated to share feedback by informing them of the benefits of planned feedback activities before inviting them to participate. The findings about adversarial interactions between HFC members and the public, linked to differing worldviews on the role of HFCs and an organisational context in which frontline providers are overworked and experience burn-out, suggests the need for relationship and capacity building among both HFC members and staff. This role could be taken up by a dedicated SCHMT member, as was the case in Kenya during pre-devolution times [55].

More broadly, economic empowerment is important to begin to address structural power imbalances. As Flores et al. argue in their exploration of social participation in Guatemala, *“inclusion of traditionally excluded groups in decision-making processes does not create agency unless there are actions or policies that improve the material conditions of that population” (pg 38)* [56]. Though economic empowerment requires wider governmental action beyond the health sector, health system actors have a role to play in calling for attention to it not only as a determinant of health, but also as an enabler of participation in feedback mechanisms among vulnerable groups. To address deep-seated subtle power manifesting in societal norms and how institutions are organised, public health managers and decisionmakers could tailor strategies for raising consciousness to specific groups and issues. For example, elections and other activities for participatory mechanisms could be scheduled at times when youth, women and other vulnerable groups can attend. Further, to encourage youth and People With Disability to participate might include shifting institutional arrangements to include multiple ways such as the use of social media to engage on a wide range of issues including policy processes such as budgeting and planning.

To address victimisation concerns, if the public perceived that they would be heard and there would be no retribution for their views, they might use available feedback mechanisms to share feedback. However, changing public perceptions of how their input is valued requires deep and sustained engagement, going beyond simple awareness creation campaigns. Further, people would need to see their input being actively considered to enhance trust in the system. This could be achieved through adapting feedback mechanisms. For example, deliberative approaches in consultation could be adopted in public participation meetings for budgeting and planning issues which in our study were characterised by contestation and were demonstrated to lack sufficient public input. Deliberative approaches require selection of participants through, for example, stratified sampling of the population to ensure diversity of representatives, providing information timeously to the public, in a manner that can be understood, and allowing room for consideration of trade-offs, two-way consultation and debate [57, 58]. A study from South Africa demonstrated that when members of the public were informed of health system resource constraints, they were able and willing to make trade-offs and to reach a consensus regarding local priorities [59].

Literature suggests that the willingness of those with hierarchical power to support implementation is an important pre-condition for successful initiatives [60, 61]. However, an organisational culture of defensiveness, as illustrated in our study and elsewhere [62, 63], is likely to significantly constrain not only responsiveness to public feedback but also, the public giving feedback in the first place. Leveraging their organisational power relationships, more powerful health system actors (such as the CHMT) can frame public feedback processes as activities that support learning and health system change. This echoes literature on complaints management which suggests that leadership commitment to a view of complaints as valuable for improvement is key to a positive impact [42, 64, 65]. Such a framing could relieve the ‘threatening’ nature of negative public feedback and reduce an organisational culture of defensiveness [65]. Finally, transparency in feedback management can bridge information asymmetry and sustain actor agency [42, 65]. In our study, SCHMTs reportedly received limited information from the County Health Management Team, weakening their ability to respond to public feedback. Enhancing the flow of information on health system decisions (and their rationale) would be a step towards building SCHMT and public agency and strengthening responsiveness.

This analysis combining Gaventa’s power cube and Long’s actor lifeworld enabled us to explore both structural forms and flow of power (Gaventa) and gain insights into power dynamics at the granular level between actors, showing who, when and how different actors’ interests impacted responsiveness (Long’s interface analysis). The two power frameworks were complementary, useful for examining the multifaceted nature of responsiveness, and generating ideas about strategies for changing power dynamics within organisations and between actors towards strengthening responsiveness to public feedback.

### Study limitations

In this work, we focused on two SCHMTs and two of their linked HFCs. Given the complexity and context-specific nature of responsiveness, the findings cannot be generalised to the population from which the cases are derived – all SCHMTs and HFCs across Kenya. However, case study work supports analytic generalizability, where conclusions about relationships between concepts can be drawn that are transferable to other settings [30, 31]. Thus, some of the learning generated from this work can support reflection on responsiveness in other settings. While actor interactions and power dynamics are important in health system responsiveness, we also recognise that there are important influences – such as existing policies, how responsiveness is framed within policy documents, and the broader political and economic context in which a health system exists.

## Conclusion

Power is mentioned in the literature on responsiveness, but rarely explored in-depth. In this study we have demonstrated that decisions and actions pertaining to public feedback across case study SCHMTs and HFCs were influenced by a complex interplay of forms and practices of power. Our exploration of power draws attention to organisational influences on health system responsiveness (within the health system and from the broader public sector). This in-depth investigation of the lived social realities of actors illuminates the political frame of organisations, as arenas for ‘*ongoing interplay of divergent interests and agendas’* (pg 234) [66]. We observed multiple power practices at one actor interface, and how one actor lifeworld underpinned positive power practices in one instance, and negative power practices in another instance. These interactions in turn had varying effects on the functioning of feedback mechanisms, inclusion of vulnerable groups, and the processes of receiving and responding to public feedback. Our analysis of HFC and SCHMT experiences support the proposition that strengthening system responsiveness requires multistakeholder interaction across multiple levels, combined with active facilitation of feedback mechanisms to be representative particularly of vulnerable groups and to function effectively as well as empowering the public to share feedback. Empowerment would be most effective when it extends beyond building individual agency to share feedback to include addressing structural power that is often subtle and difficult to see, and enabling health managers and providers through relationship building with other more powerful actors.

Our findings are relevant to health system decision-makers who develop responsiveness policies and guidelines; to health managers who interact with political actors and representatives as well as other broader public sector decision-makers involved in processes where public feedback is received into the health system; and to researchers with an interest in how public feedback is incorporated into health system decision-making. The approach to power analysis adopted in this study could be applied to empirical examinations of power in other ‘processing spaces’ in the health system, in which public feedback is received and responses generated.

### Supplementary Information

Supplementary Material 1.

Supplementary Material 2.

Supplementary Material 3.

## Data Availability

The data that support the findings of this study are available from KEMRI Wellcome Trust but restrictions apply to the availability of these data, which were used under license for the current study, and so are not publicly available. Data are however available from the authors upon reasonable request and with permission of KEMRI Wellcome Trust.
