# Supplementary material for: Power and positionality in the practice of health system responsiveness at sub-national level: insights from the Kenyan coast
Source: Int J Equity Health. 2024 Sep 2;23:177. doi: 10.1186/s12939-024-02258-5 (PMC11367973; doi:10.1186/s12939-024-02258-5)
Supplement: Supplementary file 1 — Supplementary Material 1. [file 12939_2024_2258_MOESM1_ESM.docx]

**Interview guide for CHMT/SCHMT members, also adapted for PHC facility in-charges**

**Introduction**

I would like to have a discussion with you as a manager(s) working at county/sub county/hospital/facility level and who is familiar with various ways that the public share feedback that is views, concerns and input into the health system. I would like to ask you questions about channels for giving feedback to the health system used by citizens or groups of citizens that you are aware of, how information from these feedback channels reaches decision-makers and how this information is processed and what system responses there have been. I would also like to learn how health system challenges have influenced how the health system responds to feedback/concerns/needs voiced by the community.

| **Back-ground** | -Can you tell me about your current position/role in the (county/sub-county) health system?  *Probes: For how long have you been in that position? Who do you report to? Probe for when was your SCHMT was formed? Was it formerly a sub-district prior to devolution? Or is it among the more recent ones? Other probes related to reporting: Who do you think you’re accountable to? Why?* |
| --- | --- |
| **Broad questions about the functioning of the SCHMT and related to receiving public feedback** | -What is the structure of the CHMT/SCHMT? And what roles does the CHMT /SCHMT play within the health system? In your view to what extent would you say information (concerns, complaints, compliments, priorities) from the public is incorporated into health sector plans, projects, service delivery? If low, why is this so?, If high, how has the county been able to achieve this? What are the ways/channels through which the CHMT/SCHMT receives feedback(views, concerns, complaints) from the public? (*Probe for routine collection of public and users views or more random, ad hoc. Probe also for formal-e.g. health sector stakeholder forum; multiple sector stakeholder forum, health committees etc /informal mechanisms)* Is there a person/team designated within the CHMT/SCHMT to handle community/public views, priorities, complaints, suggestions? *Who does this team comprise of? How was this team selected, by whom?*  *-For any of the mechanisms for receiving feedback mentioned probe for how they are resourced (e.g. the participatory mechanisms such as HFCs, CHSF), who is assigned to access information collected (e.g. for hotlines, suggestion boxes, surveys)*  -Are there any instances where you have received information about public views/concerns from actors higher up in the health system/outside the health system but within the public sector/elsewhere e.g. from CSOS/NGOs? *(Probe for when this has happened, how frequently, particular incident)*  *-*Which sources of information about public views/priorities do you prioritise, why? What are some of the key issues/concerns that have been raised by the public and health system users recently? Would you say there are some forms of information/views/concerns that are prioritised over others? Why is this so?  What challenges do you experience in accessing information to public feedback? |
| **Processing public feedback** | -What happens to these public/community views, priorities or concerns when they are received by a member of the CHMT/SCHMT? How do you integrate the feedback from a)multiple channels b)multiple facilities *Probe for other processing e.g. analysis, consolidation, prioritization. If any of these happens, is there a designated person/team for it? If yes, who comprises the team? Is there support for analysis, consolidation, from elsewhere e.g. from NGOs/CSOs (if yes, to what extent are they involved?)*  -Is there feedback that is escalated upwards to other health system actors, or re-directed elsewhere? How is the decision to do this made? *Are there any guidelines/framework that the SCHMT uses to determine how to handle public feedback?* |
| **Responding to public feedback** | -As part of the CHMT/SCHMT, were you able to respond to the concerns/issues/priorities you mentioned . If yes, what kinds of responses have you been able to enact? Were the public made aware of how their concerns were addressed/if their views were taken up? How? Other than the CHMT/SCHMT were there instances where engaged other actors to generate a response? Which actors were these?  *(Probe for a critical incident, and attempt to track back to a response or inaction from the health system)*  *-*For issues that get escalated upwards for action to the health system, how do you learn about whether any action has been taken? |
| - **Enablers** - **Barriers** | -How well would you say the mechanisms/channels through which you receive public views/concerns/complaints/compliments function. Why do you think they function so well (or not?)  -What would you say enables/limits you as a CHMT/SCHMT member/or CHMT/ SCHMT to receive and respond to community concerns/view |
| **-Vulnerable groups** | - Who are those people who find it difficult to seek health services in the community you work in? (Or which groups of people in the community you work in mostly need health services, but you face challenges in providing them with community health services)  - How would you say their voices are included in the information you receive from the public? *Probe for groups within the community that the respondent thinks are vulnerable but their voices are not included in feedback channels currently in use.*  Is there any particular concern/compliment/view that you learnt about concerning experiences of vulnerable groups with the health system that comes to mind? How was this responded to?  Are there any local initiatives that you are aware of which have been successful for ensuring that everyone in the community gets the community health services that they need? |
| **Health system shocks (e.g. COVID-19/HCW strikes)** | -To what extent would you say the public’s views and concerns were/have been integrated in the COVID-19 response (from the early days to date?-for example in selection of COVID-19 isolation facilities, to learn about community challenges in accessing care) *Probe for what mechanisms were used to learn about public concerns and views? Were there mechanisms for feedback that you expected would be used but were not used? Why do you think this is so? Who were the main decision-makers regarding what actions would be taken in response to public feedback/or in determining what information was released to the public? Were there any new mechanisms introduced? Who were the main actors involved in the introduction of these mechanisms? What was the SCHMT’s role (if any) during the introduction of these mechanisms? Have newly introduced mechanisms continued to function well (or not) to date?*  *-*There was a HCWs strike towards the end of 2020, and beginning of 2021. Were there any views, concerns, feedback that you recall receiving /your team received from the public at this time. |
| **Overall value of public feedback, Additional qns related to power** | To what extent would say information (concerns, complaints, compliments, priorities) from the public is incorporated into health sector plans, projects, service delivery? (*moved up to serve as introductory question*)  What opportunities exist for the public to learn about what happened with feedback they received?  Who do you think are the really important people when it comes to making decisions about feedback from the public? Please explain why you think so?  - Do you have any ideas or suggestions about what would help ensure that feedback mechanisms function well and the community receives the feedback they need? What about ideas for making system wide changes based on public feedback? |
|  |  |

FGD Guide Topics (FGDs with community members-Facility Management Committee members)

| **Introduction:** We would like to hold discussions with you as members of the community who use the health system to hear more about their experience of the health system. You were selected because you participate HFC member in this facility. Specifically, we want to learn about   - Which channels you use to give feedback to the health system - How the feedback you give is processed - If and how you get any responses on the feedback that you give - We would like you to take part in a discussion with 5-6 other HFC member - If you do not want to answer any of the questions you may say so and the interviewer will move on to the next question. - The discussion will be recorded to assist later in fully writing up the information. No-one will be identified by name in the recording but if you do not agree to be recorded we will proceed without it and take notes |
| --- |

| **Questions about formation of the HCF and roles** | -Can you tell us about your roles in the HFC?  *Probes: For how long have you been in that position? To whom are you accountable to? How was the current HFC formed? E.g. through elections, selection (by whom?) When was selection/election done last? When a HFC member leaves or is unable to continue their roles (ask about possible reasons for this) how is the vacant member position filled*  -Is there any form of training that you attend to be able to carry out your roles? Which people in the HFC attended the training? For those who were unable to attend, why was this? How could the training be changed so that more VHC members are able to attend |
| --- | --- |
| **Broad questions about the functioning of the HFC and related to receiving public feedback** | -How frequently does the HFC meet? Where are most of your meetings held? Who calls people for the meetings? Who coordinates/leads the meetings? Who keeps minutes?  -Tell me about how HFC members get along with one another (men & women, people from different villages, health system representatives)? Has there ever been an argument? What happened?  -What are the different ways that you learn about you give (or gave) feedback (or provide input) to the health system? (*Probe for ‘through, suggestion boxes, through CHWs, public participation meetings, informal mechanisms-radio, T.V. Whatsapp, other social media, AWP process, direct calls from community members, talking in the village)*.  -For those members who you felt were not represented in the HFC how do they voice their concerns, provide feedback about their experiences with the health system?  -Over the last one/two years, what kind of feedback have you received from the public and shared with health system actors? (*Probe for complaints, compliments, suggestions and on what issues) To whom did you give this feedback? (a person-CHW, Facility-in-charge, staff at the facility, MCA, a group-civil advocacy group, SCHMT member*)  -Which other groups of citizens outside the health facility (provide or have provided feedback/input to the facility? Who are the members of these groups? Do you have NGOs/CSOs in this community who help to raise public awareness regarding their health rights, and how to voice concerns about health system issues? Which are these, and in what areas have they helped the public to provide feedback on? *Probe for an example of a specific mechanism used, e.g. score-cards, public hearings etc and issues raised through these mechanisms.*  -Would you say that the community knows how and with whom to share their views about how the health system functions? If no, what do they do when they are dissatisfied with how the health system functions? Or would like to make suggestions for improvement? |
| **Processing of feedback** | - What happened to the feedback/input that you have shared with health system actors about the health system? (*Probe for what happens to for example priorities collected for annual planning, from suggestion boxes*)  -When you as member of the HFC receive input/feedback from other community members, how do you handle it? (*Probe for whether it is handled at the facilitye.g. through dialogue with other HFC members, with HFC in-charge, whether it is collated, whether it is forwarded to SCHMT, , how they decide what to forward, do they ever go directly to the CHMT or to an MCA and leave out the SCHMT*) |
| **Responding to public feedback** | - For feedback that you received as a HFC member, how did you communicate back to the public regarding a response or action that was taken? What of where action was not taken, do you also communicate that back to the public?  -For feedback that went directly to subcounty/county/national level, what responses did you get? How were the responses conveyed to you?  -As an HFC member, is there input/feedback from other community members that you have received but you did not respond to? What are some of the reasons that you did not respond to this input/feedback? For those issues that you did respond to, did the public raise them with another channel of feedback? |
| - **Enablers** - **Barriers** | -How well would you say the mechanisms/channels through which you receive public views/concerns/complaints/compliments function. Why do you think they function so well (or not?)  -What would you say enables/limits you as the HFC to receive and respond to community concerns/views |
| **-Vulnerable groups** | - Which groups do you think might have challenges in giving input/feedback to the health system?  -As an HFC member, how do you try to engage these groups for their feedback? (For example, are there specific ways for these groups to provide feedback/input?)  - How would you say their voices are included in the information you receive from the public? *Probe for groups within the community that the respondent thinks are vulnerable but their voices are not included in feedback channels currently in use.*  Is there any particular concern/compliment/view that you learnt about concerning experiences of vulnerable groups with the health system that comes to mind? How was this responded to? |
| **Health system shocks (e.g. COVID-19/HCW strikes)** | -To what extent would you say the public’s views and concerns were/have been integrated in the COVID-19 response (from the early days to date?-for example in selection of COVID-19 isolation facilities, to learn about community challenges in accessing care) *Probe for what mechanisms were used to learn about public concerns and views? Were there mechanisms for feedback that you expected would be used but were not used? Why do you think this is so? Who were the main decision-makers regarding what actions would be taken in response to public feedback/or in determining what information was released to the public? Were there any new mechanisms introduced? Who were the main actors involved in the introduction of these mechanisms? What was the HFC’s role (if any) during the introduction of these mechanisms? Have newly introduced mechanisms continued to function well (or not) to date?*  *-*There was a HCWs strike towards the end of 2020, and beginning of 2021. Were there any views, concerns, feedback that you recall receiving /your team received from the public at this time. Were you able to take any action at that time of the strike, if yes, what action, if no, why? |
| **Overall value of public feedback, Additional qns related to power** | -To what extent would say information (concerns, complaints, compliments, priorities) from the public is incorporated into health sector plans, projects, service delivery?  -What opportunities exist for the public to learn about what happened with feedback they received?  Who do you think are the really important people when it comes to making decisions about feedback from the public? Please explain why you think so?  - Do you have any ideas or suggestions about what would help ensure that feedback mechanisms function well and the community receives the feedback they need? What about ideas for making system wide changes based on public feedback?   - What are the challenges facing the HFC? What would help the HFC overcome these challenges? - What do you think needs to change to make the HFC function better? *Probe on: How would different aspects (other members, resources, training, health system, other stakeholders) need to change?* |
