# Supplementary material for: Power and positionality in the practice of health system responsiveness at sub-national level: insights from the Kenyan coast
Source: Int J Equity Health. 2024 Sep 2;23:177. doi: 10.1186/s12939-024-02258-5 (PMC11367973; doi:10.1186/s12939-024-02258-5)
Supplement: Supplementary file 3 — Supplementary Material 3. [file 12939_2024_2258_MOESM3_ESM.docx]

Supplementary material 3: Multiple actor interfaces and interconnections in relation to receiving and responding to public feedback

Abbreviations: CEC-County Executive Committee member for Health, CHC-Community Health Committee1, CHMT-County Health Management Team, CHV-Community Health Volunteer, CPSB-County Public and Service Board, HFC-Health Facility Committee, MCA-Member of County Assembly, SCHMT-Sub-county Health Management Team

Figure S1: Multiple actor interfaces and interconnections in relation to receiving and responding to public feedback

The figure in this supplementary material highlights the multiple sets of actors and interactions that impacted on responsiveness - showing the complexity of system-level responsiveness. For example, the public formed interfaces with varied actors such as their community representatives within the health system (community health volunteers and health facility committees), with local politicians (Members of the County Assembly) and with appointed senior county officials. These interactions occurred across and within Gaventa’s spaces and levels of power. Within the health system, the public interacted with health managers at county, sub-county, and facility levels to share feedback. The diagram also illustrates interactions between the public and actors in an invited space (HFC), and in a closed space (SCHMT) where public feedback was received. More detail about the closed and invited spaces is provided below. Health system actors also interacted among themselves (Figure S1, green section), and with non-health system actors (Figure S1, purple section), to generate responses. Several of these interactions influenced how feedback mechanisms functioned, and therefore impacted responsiveness, albeit indirectly. Exercises of power at these interfaces were explored in in relation to i) receiving, and ii) responding to public feedback.
